# Clinical outcomes after treatment with direct antiviral agents: beyond the virological response in patients with previous HCV-related decompensated cirrhosis

**DOI:** 10.1186/s12879-022-07076-0

**Published:** 2022-01-27

**Authors:** Georges-Philippe Pageaux, Clovis Lusivika Nzinga, Nathalie Ganne, Didier Samuel, Céline Dorival, Fabien Zoulim, Carole Cagnot, Thomas Decaens, Dominique Thabut, Tarik Asselah, Philippe Mathurin, François Habersetzer, Jean-Pierre Bronowicki, Dominique Guyader, Isabelle Rosa, Vincent Leroy, Olivier Chazouilleres, Victor de Ledinghen, Marc Bourliere, Xavier Causse, Paul Cales, Sophie Metivier, Véronique Loustaud-Ratti, Ghassan Riachi, Laurent Alric, Moana Gelu-Simeon, Anne Minello, Jérôme Gournay, Claire Geist, Albert Tran, Armand Abergel, Isabelle Portal, Louis d’Alteroche, François Raffi, Hélène Fontaine, Fabrice Carrat, Stanislas Pol, For the French ANRS CO22 Hepather Cohort, Delphine Bonnet, Virginie Payssan-Sicart, Chloe Pomes, François Bailly, Marjolaine Beaudoin, Dominique Giboz, Kerstin Hartig-Lavie, Marianne Maynard, Eric Billaud, David Boutoille, Morane Cavellec, Caroline Chevalier, Isabelle Hubert, Pierre Goepfert, Adrien Lannes, Françoise Lunel, Jérôme Boursier, Nathalie Boyer, Nathalie Giuily, Corinne Castelnau, Giovanna Scoazec, Aziza Chibah, Sylvie Keser, Karim Bonardi, Anaïs Vallet-Pichard, Philippe Sogni, Juliette Foucher, Jean-Baptiste Hiriart, Amandine Legendre, Faiza Chermak, Marie Irlès-Depé, Si Nafa Si Ahmed, Christelle Ansaldi, Nisserine Ben Amara, Valérie Oules, Jacqueline Dunette, Rodolphe Anty, Eve Gelsi, Régine Truchi, Elena Luckina, Nadia Messaoudi, Joseph Moussali, Barbara De Dieuleveult, Héloïse Goin, Damien Labarrière, Pascal Potier, Si Nafa Si Ahmed, Véronique Grando-Lemaire, Pierre Nahon, Séverine Brulé, Rym Monard, Caroline Jezequel, Audrey Brener, Anne Laligant, Aline Rabot, Isabelle Renard, Thomas F. Baumert, Michel Dofföel, Catherine Mutter, Pauline Simo-Noumbissie, Esma Razi, Hélène Barraud, Mouni Bensenane, Abdelbasset Nani, Sarah Hassani-Nani, Marie-Albertine Bernard, Georges-Philippe Pageaux, Michael Bismuth, Ludovic Caillo, Stéphanie Faure, Marie Pierre Ripault, Christophe Bureau, Sarah Launay, Jean Marie Peron, Marie Angèle Robic, Léa Tarallo, Marine Faure, Bruno Froissart, Marie-Noelle Hilleret, Jean-Pierre Zarski, Odile Goria, Victorien Grard, Hélène Montialoux, Muriel François, Christian Ouedraogo, Christelle Pauleau, Anne Varault, Tony Andreani, Bénédicte Angoulevant, Azeline Chevance, Lawrence Serfaty, Teresa Antonini, Audrey Coilly, Jean-Charles Duclos Vallée, Mariagrazia Tateo, Corinne Bonny, Chanteranne Brigitte, Géraldine Lamblin, Léon Muti, Abdenour Babouri, Virginie Filipe, Camille Barrault, Laurent Costes, Hervé Hagège, Soraya Merbah, Paul Carrier, Maryline Debette-Gratien, Jérémie Jacques, Guillaume Lassailly, Florent Artu, Valérie Canva, Sébastien Dharancy, Alexandre Louvet, Marianne Latournerie, Marc Bardou, Thomas Mouillot, Yannick Bacq, Didier Barbereau, Charlotte Nicolas, Caroline Chevalier, Isabelle Archambeaud, Sarah Habes, Nisserine Ben Amara, Danièle Botta-Fridlund, Eric Saillard, Marie-Josée Lafrance, Carole Cagnot, Alpha Diallo, Lena Wadouachi, Ventzi Petrov-Sanchez, Douae Ammour, Loubna Ayour, Jaouad Benhida, Fabrice Carrat, Frederic Chau, Céline Dorival, Audrey Gilibert, Isabelle Goderel, Warda Hadi, Clovis Luzivika Nzinga, Grégory Pannetier, François Pinot, Odile Stahl, François Téloulé

**Affiliations:** 1 Department of Hepatology and Gastroenterology, Centre Hospitalo-Universitaire Saint Eloi, Université de Montpellier, 34295 Montpellier, France; 2 Sorbonne Université, Institut National de la Santé et de la Recherche Médicale, Institut Pierre Louis d’Epidémiologie et de Santé Publique, Paris, France; 3 Department of Hepatology, Hôpitaux Universitaires Paris Seine-Saint-Denis, Site Avicenne, AP-HP, Bobigny, France; 4 Université Paris 13, Sorbonne Paris Cité et INSERM UMR 1162, Paris, France; 5 AP-HP Hôpital Paul-Brousse, Centre Hépato-Biliaire, 94800 Villejuif, France; 6 Université Paris-Saclay, Inserm, Physiopathogénèse et Traitement des Maladies du Foie, 94800 Villejuif, France; 7 Inserm, Unité 1193, Université Paris-Saclay, Hepatinov, 94800 Villejuif, France; 8 Department of Hepatology, Hospices Civils de Lyon, INSERM U1052, Université de Lyon, Lyon, France; 9 Unit for Basic and Clinical Research on Viral Hepatitis, ANRS (France REcherche Nord&Sud Sida-Vih Hépatites), Paris, France; 10 Université Grenoble Alpes, 38000 Grenoble, France; 11 Institute for Advanced Biosciences, Research Center Inserm U1209, CNRS UMR5309, 38700 La Tronche, France; 12 Service d’hépatogastroentérologie, Pôle Digidune, CHU Grenoble Alpes, 38700 La Tronche, France; 13 Department of Hepatology and Gastroenterology, Groupe Hospitalier Pitié-Salpétrière, AP-HP, INSERM UMR-S938, Sorbonne Université, Paris, France; 14 INSERM UMR 1149, Hepatology, Hospital Beaujon, Centre de Recherche sur l’Inflammation, (CRI), University Paris Diderot, Clichy, France; 15 Service des Maladies de l’appareil Digestif, Université Lille 2 and Inserm U795, Lille, France; 16 CIC, Inserm 1110 et Pôle Hépato-digestif des Hôpitaux Universitaires de Strasbourg, Université de Strasbourg, Strasbourg, France; 17 Inserm U1254 and Department of Hepato-Gastroenterology, University Hospital of Nancy Brabois, Université de Lorraine, Vandoeuvre-les-Nancy, France; 18 CHU de Rennes, Service d’hépatologie, 35033 Rennes, France; 19 Univ Rennes1, Inra, Inserm, Institut NUMECAN (Nutrition, Métabolismes et Cancer), UMR_A 1341, UMR_S 1241, 35033 Rennes, France; 20 Department of Hepatology and Gastroenterology, Centre Hospitalier Intercommunal, Créteil, France; 21 Department of Hepatology and Gastroenterology, Hôpital Henri Mondor, AP-HP, Université Paris-Est, INSERM U955, Créteil, France; 22 Department of Hepatology, Hôpital Saint-Antoine, AP-HP, Sorbonne Université, Paris, France; 23 Hepatology Unit Hôpital Haut-Lévêque, Pessac, INSERM U1053, Université Bordeaux Segalen, Bordeaux, France; 24 Department of Hepatology and Gastroenterology, Hôpital Saint Joseph, Marseille, France; 25 Department of Hepatology and Gastroenterology, CHR Orléans, Orléans, France; 26 Hepatology Department, University Hospital, Angers, France; 27 HIFIH Laboratory, Angers University, Angers, France; 28 Hepatology Unit, CHU Rangueil, 31059 Toulouse, France; 29 Department of Hepatology and Gastroenterology, CHU Limoges, U1248 INSERM, Univ. Limoges, 87000 Limoges, France; 30 Department of Hepatology and Gastroenterology, CHU Charles Nicolle, Rouen, France; 31 Department of Internal Medicine and Digestive Diseases, CHU Purpan, UMR 152 Pharma Dev, IRD Toulouse 3 University, Toulouse, France; 32 Service d’Hépato-Gastroentérologie, CHU de la Guadeloupe-Faculté de Médecine, Université des Antilles, 97110 Pointe-à-Pitre Cedex, France; 33 INSERM, UMR-S1085/IRSET, 35043 Rennes, France; 34 Department of Hepatology and Gastroenterology, University Hospital Dijon, INSERM UMR 1231, Dijon, France; 35 Gastroenterology and Hepatology Department, Institut des Maladies de l’Appareil Digestif, University Hospital of Nantes, Nantes, France; 36 Department of Hepatology and Gastroenterology, Centre Hospitalier Régional, Metz, France; 37 Digestive Center, Centre Hospitalier Universitaire de Nice, INSERM U1065-8, Nice, France; 38 Department of Digestive and Hepatobiliary Diseases, Estaing University Hospital, Clermont-Ferrand, France; 39 UMR 6602 CNRS-Sigma-Université Clermont Auvergne, Clermont‐Ferrand, France; 40 Service d’Hépato-Gastroentérologie, Hôpital de la Timone, Aix-Marseille Université, AP-HM, Marseille, France; 41 Unit of Hepatology, Hépatogastroentérologie, CHU Trousseau, 37044 Tours, France; 42 Department of Infectious Diseases, Hotel-Dieu Hospital-INSERM CIC 1413, Nantes University Hospital, Nantes, France; 43 Assistance Publique-Hôpitaux de Paris, Hôpital Cochin, Unité d’Hépatologie, Paris, France; 44 Assistance Publique-Hôpitaux de Paris, Hôpital Saint-Antoine, Unité de Santé Publique, Paris, France; 45 Université Paris Descartes, Paris, France; 46 INSERM U-1223 et USM20, Institut Pasteur, Paris, France

**Keywords:** Hepatitis C virus, Decompensated cirrhosis, Direst-acting antiviral agents, Survival, Hepatocellular carcinoma, Sustained virological response

## Abstract

**Background:**

In HCV-infected patients with advanced liver disease, the direct antiviral agents-associated clinical benefits remain debated. We compared the clinical outcome of patients with a previous history of decompensated cirrhosis following treatment or not with direct antiviral agents from the French ANRS CO22 HEPATHER cohort.

**Methods:**

We identified HCV patients who had experienced an episode of decompensated cirrhosis. Study outcomes were all-cause mortality, liver-related or non-liver-related deaths, hepatocellular carcinoma, liver transplantation. Secondary study outcomes were sustained virological response and its clinical benefits.

**Results:**

559 patients met the identification criteria, of which 483 received direct antiviral agents and 76 remained untreated after inclusion in the cohort. The median follow-up time was 39.7 (IQR: 22.7–51) months. After adjustment for multivariate analysis, exposure to direct antiviral agents was associated with a decrease in all-cause mortality (HR 0.45, 95% CI 0.24–0.84, p = 0.01) and non-liver-related death (HR 0.26, 95% CI 0.08–0.82, p = 0.02), and was not associated with liver-related death, decrease in hepatocellular carcinoma and need for liver transplantation. The sustained virological response was 88%. According to adjusted multivariable analysis, sustained virological response achievement was associated with a decrease in all-cause mortality (HR 0.29, 95% CI 0.15–0.54, p < 0.0001), liver-related mortality (HR 0.40, 95% CI 0.17–0.96, p = 0.04), non-liver-related mortality (HR 0.17, 95% CI 0.06–0.49, p = 0.001), liver transplantation (HR 0.17, 95% CI 0.05–0.54, p = 0.003), and hepatocellular carcinoma (HR 0.52, 95% CI 0.29–0.93, p = 0.03).

**Conclusion:**

Treatment with direct antiviral agents is associated with reduced risk for mortality. The sustained virological response was 88%. Thus, direct antiviral agents treatment should be considered for any patient with HCV-related decompensated cirrhosis.

*Trial registration*: ClinicalTrials.gov registry number: NCT01953458.

**Supplementary Information:**

The online version contains supplementary material available at 10.1186/s12879-022-07076-0.

## Background

The 2000s were marked by an increase in the prevalence of cirrhosis and hepatocellular carcinoma (HCC), mainly due to hepatitis C virus (HCV) infection: 48% of cirrhosis deaths and 67% of HCC deaths in 2013 [1]. At the same time, HCV infection was the primary indication for liver transplantation in western countries, with over 20% of all LT candidates on the waiting list having HCV infection [2]. Direct-acting antiviral agents (DAAs) have transformed the clinical course of HCV-infected patients, even in those with advanced liver disease [3–5]. In most studies, the primary end-point has been sustained virological response (SVR) at 12 weeks after the end of therapy, and the secondary end-point logically being dedicated to safety. The clinical benefit of treatment has been assessed at large by the evolution of Child–Pugh and MELD prognostic scores during a short period from baseline to post-treatment week 12, as well as by patient delisting from liver transplant waiting lists. However, the DAA-associated benefits, including the reduction of mortality or other hepatic complications, remain debated [6]. Given it is ethically difficult to design a DAA versus placebo study to investigate this issue, observational multicenter cohorts with prospective data collection, including both treated and untreated HCV-infected patients, with a significant follow-up are more relevant. The most valuable population consists of patients with decompensated cirrhosis and a short- or medium-term vital prognosis, especially if the cause of the liver disease (i.e. HCV infection) is not controlled. Among the various studies published on the evaluation of the efficacy of DAA in the most severe patients, decompensated cirrhosis has been defined either by: (1) Child–Pugh score > B7 without taking complications into account, or (2) a past or current clinical event reflecting decompensation, such as ascites, digestive haemorrhage, and encephalopathy.

The aim of this study was to compare the clinical outcome of patients with a previous history of decompensated cirrhosis following treatment or not with DAAs from the French ANRS CO22 HEPATHER cohort.

## Methods

### Study design and participants/procedures

The ANRS CO22 HEPATHER cohort, “Therapeutic Option for Hepatitis B and C: A French Cohort”, is a national, multicentre, prospective, observational cohort study of patients with viral hepatitis B or C that started in August 2012 (ClinicalTrials.gov registry number: NCT01953458). The main initial objectives of this study were to quantify the clinical efficacy and safety of new hepatitis treatments in real-life. The details of the cohort have been previously specified (see references [7] and [8] for a complete description). This study was observational and the choice of treatment combination, treatment timing, and screening for HCC or the progression of fibrosis was granted by the physician. Nevertheless, national French recommendations based on the European Association for the Study of the Liver (EASL) guidelines [9] were followed. Written informed consent was obtained from each patient before study enrolment. The protocol was performed in accordance with the Declaration of Helsinki and the French law for biomedical research. It was approved by the "CPP Ile de France 3" Ethics Committee (Paris, France) and the French Regulatory Authority (ANSM).

Among the HCV patients included in this cohort between August 2012 and December 2015, we identified those who had experienced an episode of decompensated cirrhosis before or at the time of study inclusion, including ascites, jaundice, encephalopathy, and haemorrhage. The time since decompensation was registered. We excluded patients with a history of HCC and those who had undergone LT. Patients were classified into 2 groups according to DAA exposure. As HEPATHER is an observational cohort, the decision to treat or not was at the discretion of each investigator. The potential predictors of clinical outcome assessed at study inclusion were: socio-demographics, HCV history, severity of liver disease according to Child–Pugh and MELD scores, and co-morbidities (i.e. diabetes, arterial hypertension (AHT), alcohol use, and history of HCC). A separate analysis was carried out in patients with a MELD score > 20 and/or Child–Pugh score of C, hence patients classified with severe disease.

### Outcomes

Study outcomes were all-cause mortality, subsequently classified into liver-related (LR) or non-liver-related (NLR) deaths, HCC incidence, and need for LT. The causes of death were classified by an adjudication committee comprised of two hepatologists (HF and MB) and one methodologist (CD). Adjudication was based on medical records and investigators completed a specific case report form. Data on HCC incidence included the number of lesions at diagnosis, the largest nodule size, total size, diagnostic imaging procedures, and treatment. Secondary study outcomes were SVR in the group of patients exposed to DAAs and clinical outcomes in patients with severe disease.

### Statistical analysis

Survival time was calculated as the time between study inclusion (unexposed period) or the start of first treatment (exposed period), and the last follow-up visit, outcome date (death, HCC or LT), or April 1st 2019 (the first occurring of these events). Baseline characteristics were compared using the Mann–Whitney test for quantitative variables or the Fisher’s exact test for categorical variables. Incidence rates and 95% confidence intervals (CIs) were calculated by an exact method based on a Poisson distribution.

The propensity of receiving DAA or not at study inclusion was estimated by a logistic regression model including covariates evaluated at study inclusion with dummy indicators for missing covariate values. The logistic regression model included age, gender, geographic origin, body mass index (BMI), AHT, diabetes, fibrosis score, HCV treatment-naive, HCV genotype, current excessive alcohol consumption, past excessive alcohol consumption, serum albumin level, prothrombin rate, platelet count, alanine aminotransferase (ALT) level, aspartate aminotransferase (AST) level, and alpha-fetoprotein (AFP) level. The inverse probability of treatment weighting (IPTW) analysis was used. Stabilised weights were calculated and the balance of baseline covariates was assessed between groups in the weighted sample. We used an IPTW Cox proportional-hazards model with exposure to treatment modeled as a time-varying covariate. To avoid immortal time bias, IPTW Kaplan–Meier curves were generated using the clock-reset approach for patients exposed to DAA during follow-up. Univariate and multivariate-adjusted Cox proportional-hazards models were estimated and departures from the proportionality assumption were checked by using the Schoenfeld residuals. Hazard ratios with 95% CIs for the different outcomes were estimated using competing risk analysis with cause-specific hazards. Cumulative incidence functions were estimated by the Gray’s test. The Gray’s test was used to compare the cumulative incidences between treated and untreated patients. Categorisation of continuous covariates was based on previously determined clinically relevant thresholds (all biological parameters) or quartile divisions. Missing covariate values were handled using indicators for missing data in the multivariate model. To better characterise the potential effects of SVR in patients exposed to DAAs compared with untreated patients, the exposure period was divided into: (1) on-treatment period (from the first to last day of DAA treatment, extended for 3 months), and (2) the period with a measurable SVR status (from 3 months after the last day of DAA treatment to the end of follow-up). These were regarded as time-dependent covariates in the Cox models. All analyses were performed with SAS 9.4 (SAS Institute Inc., Cary, North Carolina, USA). A p-value < 0.05 was considered as statistically significant.

## Results

Among the 14,657 HCV mono-infected patients included in this cohort, there were 4404 patients with cirrhosis, including 769 patients with a history of decompensation. Among these, 559 were eligible for study inclusion. Among these 559 patients there were 55 patients with severe liver disease with a Child–Pugh score of C and/or MELD score > 20.

DAA treatment began for 483 patients after a median time from study entry of 0.9 [0–6.2] months. At the last follow-up visit, 76 patients (13.6%) remained untreated. The median follow-up time was 39.7 (Interquartile Range (IQR): 22.7–51) months. Baseline characteristics according to DAA exposure during follow-up are shown in Table 1. Patients having received DAA in comparison with untreated patients had a less severe liver disease according to Child–Pugh score (score A, 57% vs 37%, p = 0.003) and MELD score (score < 13, 70% vs 51%, p = 0.003). Patients treated with DAA also had less excessive alcohol use (2% vs 9%, p = 0.001) and were more often infected with HCV genotype 3 (21% vs 15%, p = 0.01). The balance of baseline characteristics following IPTW analysis is presented in supplementary materials (Additional file 1: Table S1).

**Table 1:** Baseline characteristics of 559 patients with a history of decompensated cirrhosis

	Received DAA after study inclusion (n = 483)	Did not receive DAA after study inclusion (n = 76)	P-value
Follow-up time in months			
Median (Q1–Q3)	43.5 [26.6–52.4]	15.2 [6.2–39.2]	** < 0.0001**
Age in years			
Median (Q1–Q3)	56.6 [51.2–63.8]	56.0 [51.2–63.1]	0.73
Male gender	336 (70%)	47 (62%)	0.18
BMI (kg/m^2^)			0.10
< 18.5	12 (2%)	6 (8%)	
[18.5, 25]	229 (48%)	36 (47%)	
[> 25, 30]	164 (34%)	23 (30%)	
> 30	77 (16%)	11 (14%)	
Missing	1	0	
Geographic origin			0.95
Asia	7 (1%)	1 (1%)	
Eastern Europe	22 (5%)	3 (4%)	
France	296 (61%)	46 (61%)	
North Africa	81 (17%)	15 (20%)	
Other	60 (12%)	10 (13%)	
Sub-Saharan Africa	17 (4%)	1 (1%)	
Infection route			
Injecting drug use	145 (30%)	32 (43%)	
Transfusion	136 (28%)	19 (26%)	
Other or unknown	202 (42%)	23 (31%)	
Missing	0	2	0.06
Time since HCV diagnosis in years			0.22
Median (Q1–Q3)	14.2 [7.8–19.9]	15.1 [5.3–18.5]	
Missing	9	5	
HCV treatment history			0.07
Treatment-experienced	330 (68%)	44 (58%)	
Treatment-naive	153 (32%)	32 (42%)	
HCV genotype			0.19
1	283 (59%)	44 (62%)	
2	23 (5%)	5 (7%)	
3	102 (21%)	11 (15%)	
4	65 (14%)	8 (11%)	
5/6/7	5 (1%)	3 (4%)	
Missing	5	5	
Child–Pugh score			**0.003**
A	233 (57%)	20 (37%)	
B	150 (37%)	25 (46%)	
C	26 (6%)	9 (17%)	
Missing	74	22	
MELD score			**0.003**
< 13	335 (70%)	37 (51%)	
[13; 20]	117 (25%)	31 (42%)	
> 20	25 (5%)	5 (7%)	
Missing	6	4	
Diabetes			0.53
No	372 (77%)	61 (80%)	
Yes	111 (23%)	15 (20%)	
Arterial hypertension			0.55
No	328 (68%)	49 (64%)	
Yes	155 (32%)	27 (36%)	
Anaemia			0.11
No	338 (70%)	45 (61%)	
Yes	144 (30%)	29 (39%)	
Missing	1	2	
Albumin (g/L)			0.20
Median (Q1–Q3)	35.4 (30.8–39.8)	34 (29–38.6)	
Missing	10		
Prothrombin time (%)			0.06
Median (Q1–Q3)	74.5 (62–87)	70 (56–81)	
Missing	11	5	
Platelet count (/µL)			0.58
Median (Q1–Q3)	93,000 (64,000–136,000)	91,000 (61,000–148,000)	
Missing	10	1	
Alanine aminotransferase (UI/L)			** < 0.0001**
Median (Q1–Q3)	60 (41–95)	43 (28–61)	
Missing	4	3	
Aspartate aminotransferase (UI/L)			0.09
Median (Q1–Q3)	78 (53–111)	63.5 (39–108)	
Missing	4	2	
Alpha-fetoprotein (ng/mL)			0.40
Median (Q1–Q3)	7 (3.9–13.3)	6 (3.8–11.1)	
Missing	49	9	
Bilirubin (mg/L)			0.08
Median (Q1–Q3)	21 (13.6–32)	25.5 (14–44)	
Missing	8	1	
Past excessive alcohol use			0.23
No	252 (52%)	34 (45%)	
Yes	231 (48%)	42 (55%)	
Excessive alcohol use at study inclusion			**0.001**
No	383 (98%)	52 (91%)	
Yes	6 (2%)	5 (9%)	
Missing	94	19	
Smoking			**0.01**
No	278 (58%)	32 (42%)	
Yes	205 (42%)	44 (58%)	
Time since decompensation of cirrhosis in months			0.67
Med [IQR]	27.1 [7.4–95.7]	21 [4.8–84.0]	
Missing	10	4	
Type of decompensated cirrhosis			
Ascites	159 (36%)	24 (36%)	
Icterus	113 (25%)	20 (30%)	
Encephalopathy	36 (8%)	5 (8%)	
Haemorrhage	136 (31%)	17 (26%)	

Values in bold correspond to significant differences between the 2 groups (p < 0.05)

The median follow-up time was 39.7 [IQR 22.7–51.0] months. DAA treatment began for 483 patients after a median time from study inclusion of 0.9 [0–6.2] months

### Mortality

**Fig. 1:**
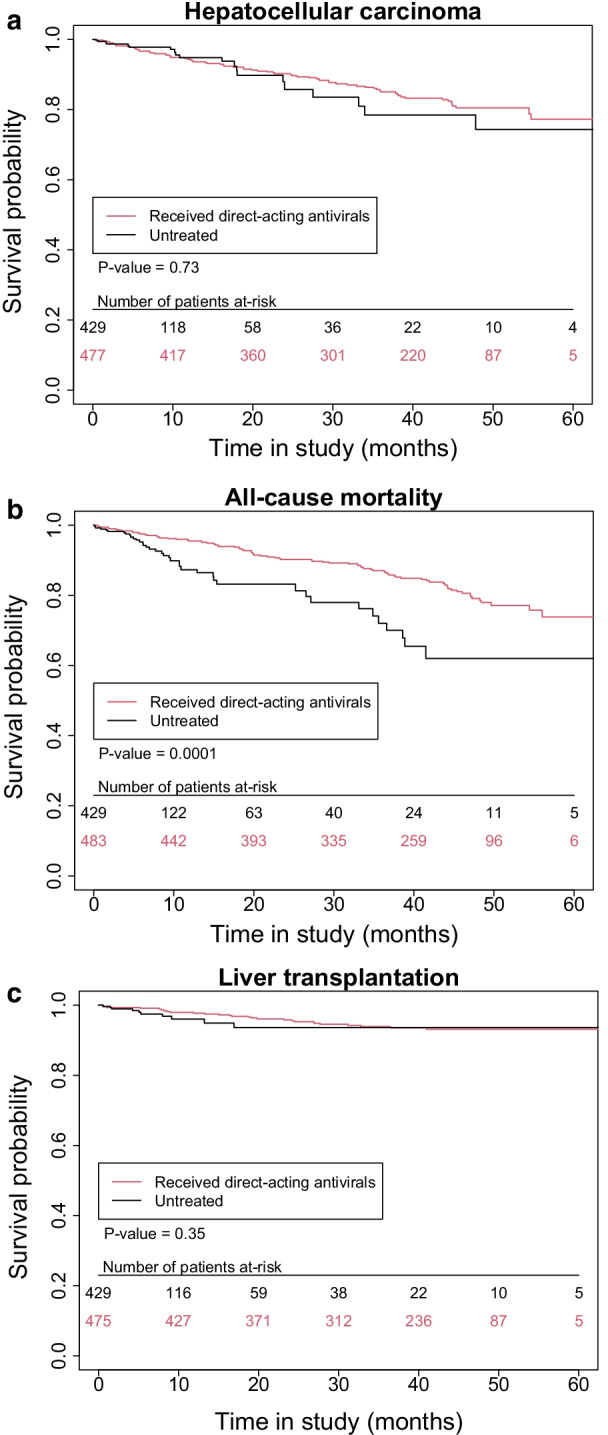
**a** Survival using inverse probability of treatment weighting (IPTW)-based analysis of hepatocellular carcinoma for all patients under study. **b** Survival using inverse probability of treatment weighting (IPTW)-based analysis of all-cause mortality for all patients under study. **c** Survival using inverse probability of treatment weighting (IPTW)-based analysis of liver transplantation for all patients under study

**Fig. 2:**
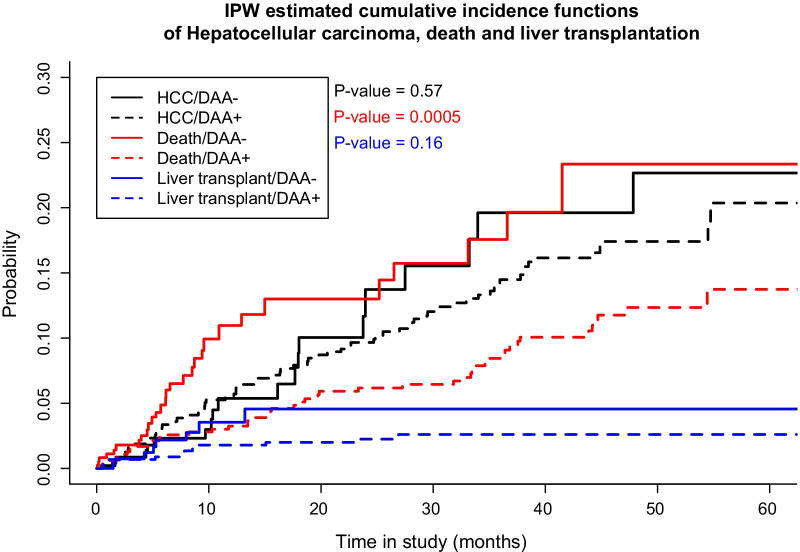
Inverse probability of treatment weighting (IPTW)-adjusted cumulative incidence functions of hepatocellular carcinoma, all-cause mortality, and liver transplantation in all patients under study. *HCC* hepatocellular carcinoma, *DAA* direct-acting antiviral, *IPW* inverse probability of treatment weighting

Out of the 559 patients included for study, 119 (21.3%) died during follow-up (80 treated and 39 untreated); 74 were classified as LR deaths, 35 as NLR deaths, and 10 were unclassified (Additional file 1: Tables S2 and S3). The incidence rates of all-cause mortality, LR deaths, and NLR deaths were higher among unexposed patients than among patients exposed to DAAs (Table 2). In the unadjusted Cox model, exposure to DAAs was associated with a decrease in all-cause mortality (HR 0.40, 95% CI 0.24–0.65, p = 0.0002) and NLR death (HR 0.35, 95% CI 0.16–0.75, p = 0.01), but not in LR death (HR 0.50, 95% CI 0.24–1.05, p = 0.07). The same findings were observed after adjustment for multivariate (Table 2) and IPTW (Table 2, Figs. 1a–c, 2) analyses. There was a significant decrease in all-cause mortality (HR 0.45, 95% CI 0.24–0.84, p = 0.01 and HR 0.42, 95% CI 0.25–0.68, p = 0.0006, respectively) and NLR death (HR 0.26, 95% CI 0.08–0.82, p = 0.02 and HR 0.34, 95% CI 0.16–0.74, p = 0.01, respectively), but not in LR death (HR 0.89, 95% CI 0.35–2.26, p = 0.81 and HR 0.60, 95% CI 0.28–1.29; p = 0.19, respectively). No other predictors other than DAA exposure were independently associated with risk for all-cause mortality (Additional file 1: Table S4).

**Table 2:** Incidence rates and hazard ratios according to DAA treatment exposure for all-cause mortality, liver-related mortality, non-liver-related mortality, and liver transplant in all 559 patients with history of decompensated cirrhosis

	Not exposed to DAA (N = 76)		Exposed to DAA (N = 483)		Unadjusted	Multivariate analysis	IPTW
	n/pyr	Incidence/100pyrs (95% CI)	n/pyr	Incidence/100pyrs (95% CI)	HR [95% CI]	HR [95% CI]	HR [95% CI]
HCC (N = 92)	20/325	6.1 (3.8–9.5)	72/1374	5.2 (4.1–6.6)	0.81 (0.47–1.38)	0.80 (0.44–1.45)	0.85 (0.50–1.47)
All cause-mortality (N = 119)	39/341	11.4 (8.1–15.6)	80/1494	5.3 (4.2–6.7)	0.40 (0.24–0.65)*	0.45 (0.24–0.84)*	0.42 (0.25–0.68)*
Liver related mortality (N = 74)	22/341	6.4 (4.0–9.8)	52/1494	3.5 (2.6–4.6)	0.50 (0.24–1.05)	0.89 (0.358–2.26)	0.60 (0.28–1.29)
Non-liver related mortality (N = 35)	11/341	3.2 (1.6–5.8)	24/1494	1.6 (1.0–2.4)	0.35 (0.16–0.75)*	0.26 (0.08–0.82)*	0.34 (0.16–0.74)*
Liver transplant (N = 36)	10/329	3.0 (1.5–5.6)	26/1414	1.8 (1.2–2.7)	0.51 (0.20–1.31)	0.53 (0.14–1.96)	0.54 (0.21–1.41)

*Significant analysis associations at the p < 0.05 level

Among the 55 patients with severe disease, 20 patients died (10 treated and 10 untreated). The incidence rate of all-cause mortality was higher in unexposed patients than in patients exposed to DAAs (Table 3). The number of events were insufficient for performance of multivariate analysis.

**Table 3:** Incidence rates according to exposure to DAA treatment for HCC, all-cause mortality, and liver transplant in all 55 patients with a Meld score > 20 or Child–Pugh score C

	Not exposed to DAA N = 12		Exposed to DAA N = 43		P-value
	n/pyr	Incidence/100pyrs (95% CI)	n/pyr	Incidence/100pyrs (95% CI)	
HCC (N = 11)	7/22	31.2 (12.5–64.3)	4/100	4.0 (1.1–10.3)	0.001
All-cause mortality (N = 20)	10/26	38.5 (18.5–70.8)	10/112	9.0 (4.3–16.5)	0.002
Liver related mortality (N = 13)	6/26	23.1 (8.5–50.2)	7/112	6.3 (2.5–12.9)	0.026
Non-liver related mortality (N = 5)	2/26	7.7 (0.9–27.8)	3/112	2.7 (0.6–7.9)	0.275
Liver transplant (N = 9)	4/20	20.0(5.5–51.3)	5/92	5.4 (1.8–12.7)	0.07

### Hepatocellular carcinoma

Ninety-two cases (72 treated and 20 untreated) of HCC were reported. In the unadjusted Cox model, exposure to DAAs was not associated with a decrease in HCC (HR 0.81, 95% CI 0.47–1.38, p = 0.43) (Table 2). The same finding was observed after adjustment for multivariate (HR 0.80, 95% CI 0.44–1.45, p = 0.46) and IPTW analyses (HR 0.85, 95% CI 0.50–1.47, p = 0.57) (Table 2, Fig. 2). Other predictors independently associated with risk for HCC were AFP, prothrombin time, and HCV genotype 3 (Additional file 1: Table S4).

Among the 55 patients with severe disease, 11 patients (7 treated and 4 untreated) presented HCC. The HCC incidence rate was higher in unexposed patients compared to patients exposed to DAAs (Table 3). There were insufficient events for carrying out multivariate analysis.

Detailed characteristics of HCC are reported in Additional file 1: Table S5. No differences were found between these patients treated with DAAs and untreated patients in terms of tumor characteristics and aggressiveness, AFP, and time between last normal imaging evaluation and diagnosis of HCC.

### Liver transplantation

Thirty-six patients (26 treated and 10 untreated) underwent LT. In the unadjusted Cox model, exposure to DAAs was not associated with a decrease in LT (HR 0.51, 95% CI 0.20–1.31, p = 0.16). The same results were detected after adjustment for multivariate (HR 0.53, 95% CI 0.14–1.96, p = 0.34) and IPTW (HR 0.54, 95% CI 0.21–1.41, p = 0.21) analyses (Table 2, Fig. 2). Prothrombin time was the only predictor independently associated with risk for LT (Additional file 1: Table S4).

Among the 55 patients with severe disease, 9 patients (5 treated and 4 untreated) underwent LT. The incidence rate of LT tended to be higher in unexposed patients (20.0 per 100 patients year) than in patients exposed to DAAs (5.4 per 100 patients year) (Table 3). There were insufficient events to perform multivariate analysis.

### SVR

Among the 444 patients who received DAAs and for whom virological data was available, the SVR12 was 88%. Among these patients, 38 presented severe disease with a SVR12 of 87%. While 12 "severe" patients remained untreated, 41 were treated with a sofosbuvir-including regimen (with daclatasvir for 25 patients, ledipasvir for 10 patients, simeprevir for 2 patients, and glecaprevir/pibrentasvir for 1 patient), with (n = 22) or without (n = 19) ribavirin. Two patients were treated with ombitasvir/paritaprevir/ritonavir. The incidence rates in treated versus untreated patients for HCC, all-cause mortality, LR mortality, NLR mortality, and LT according to DAA-exposure period and virological response status are reported in supplementary Table 6.

According to adjusted multivariable analysis, SVR achievement among treated patients was associated with a decrease in all-cause mortality (HR 0.29, 95% CI 0.15–0.54, p < 0.0001), LR mortality (HR 0.40, 95% CI 0.17–0.96, p = 0.04), NLR mortality (HR 0.17, 95% CI 0.06–0.49, p = 0.001), and LT (HR 0.17, 95% CI 0.05–0.54, p = 0.003) or HCC (HR 0.52, 95% CI 0.29–0.93, p = 0.03) (Table 4). On the contrary, not achieving a SVR was not associated with a decrease in all-cause mortality (HR 0.66, 95% CI 0.19–2.28, p = 0.52), LT (HR 0.36, 95% CI 0.07–1.82, p = 0.22), or HCC (HR 1.16, 95% CI 0.52–2.60, p = 0.72).

**Table 4:** Hazard ratios for hepatocellular carcinoma, all-cause mortality, liver-related mortality, non-liver-related mortality, and liver transplant. 483 DAA-exposed versus 76 unexposed patients are compared according to exposure period and virological response status

	On treatment N = 483		SVR N = 391		No SVR N = 53		Unknown SVR N = 39	
	Univariable HR (95% CI)	Multivariable-adjusted HR (95% CI)	Univariable HR (95% CI)	Multivariable-adjusted HR (95% CI)	Univariable HR (95% CI)	Multivariable-adjusted HR (95% CI)	Univariable HR (95% CI)	Multivariable-adjusted HR (95% CI)
Hepatocellular carcinoma	0.76 (0.45–1.29)	0.77 (0.43–1.36)	0.49 (0.28–0.85)*	0.52 (0.29–0.93)*	1.53 (0.74–3.18)	1.16 (0.52–2.60)	0.50 (0.15–1.71)	0.50 (0.15–1.73)
All-cause mortality	0.35 (0.21–0.57)*	0.47 (0.25–0.86)*	0.22 (0.13–0.37)*	0.29 (0.15–0.54)*	0.38 (0.13–1.09)	0.66 (0.19–2.28)	0.47 (0.17–1.33)	0.94 (0.28–3.11)
Liver-related mortality	0.44 (0.21–0.91)*	0.61 (0.25–1.47)	0.29 (0.14–0.62)*	0.40 (0.17–0.96)*	0.42 (0.09–1.98)	0.77 (0.15–4.10)	0.55 (0.12–2.48)	0.68 (0.14–3.43)
Non-liver-related mortality	0.30 (0.14–0.66)*	0.26 (0.09–0.76)*	0.20 (0.09–0.47)*	0.17 (0.06–0.49)*	0.24 (0.03–1.73)	0.18 (0.02–1.28)	0.57 (0.13–2.50)	1.38 (0.19–10.10)
Liver transplant	0.50 (0.20–1.25)	0.32 (0.09–1.08)	0.23 (0.08–0.67)*	0.17 (0.05–0.54)*	0.51 (0.06–4.09)	0.36 (0.07–1.82)	ND	ND

*Significant analysis associations at the p < 0.05 level. ND: Not performed due to insufficient number of events

## Discussion

To our knowledge, this study is among the first prospectively assessing the clinical outcomes of DAA-treated versus untreated HCV-infected cirrhotic patients having experienced an episode of decompensated cirrhosis. Regardless of the virological efficacy of DAAs, all-cause mortality and NLR mortality were decreased in treated patients, but not LT use or the risk of HCC. However, in DAA-treated patients achieving SVR, all-cause mortality, LR and NLR mortality, the use of LT, and the risk of HCC were decreased. These findings warrant several comments.

In decompensated cirrhotic patients it is standard to correlate the risk of mortality and the use of LT with Child–Pugh and MELD prognostic scores. Pivotal studies demonstrating the efficacy of DAAs in decompensated cirrhotic patients have shown that 12 weeks after the end of treatment, approximately 50% of patients demonstrate an improvement in Child–Pugh score and/or MELD score, thus indicating the clinical benefit of DAA treatment [3, 4]. As reported by Foster and colleagues [5], it has been suggested that this benefit was more frequently observed in young patients with mildly impaired liver function at baseline and normal natremia [5]. Although not a prospective cohort, this real-life study has some similarities with ours; there is a control group of untreated patients. It is also interesting to note in this study that while a better functional outcome based on the evolution of MELD score and the development of decompensating episodes was observed in treated patients, there were no differences in terms of mortality, HCC, or LT. This better functional outcome was considerable in patients who achieved SVR, but again without major clinical improvement. Our results obtained here over a longer period following treatment are relatively different. We observed a lower risk for all-cause mortality, particularly for NLR mortality in patients treated with DAAs compared with untreated patients. HCV-induced NLR mortality is well established and has been reported in many studies [9]. The most frequently reported causes are cancers, cardiac disorders, and renal disorders. A decrease in NLR mortality has been reported after interferon-based therapy in patients with SVR [10, 11]. The distinction between LR and NLR mortality is based on international classifications (MedDRA v17.0 classification) by an adjudication panel. Nevertheless, misclassification is always possible. For example, a car accident may be linked to a sub-clinical hepatic encephalopathy, and infections are a direct consequence of the cellular immunodepression of decompensated cirrhotic patients. Among the other possibilities explaining the absence of a significant association between DAA treatment and LR mortality in our study, of particular note is the limited number of events and the relatively short follow-up after SVR. However, it is reassuring that the LR mortality hazard ratios followed the same trend and were consistent (although not significant) with those estimated for NLR mortality. Moreover, it is also likely that a reduced risk of LR death does not occur in the very first years after SVR. This is due to the time it requires for liver restoration and improvement of cirrhosis in these patients with decompensated cirrhosis [12]. This is the reason we believe that all-cause mortality is more representative of the benefit of DAA treatment.

The persistence of the risk of developing HCC after viral eradication in cirrhotic patients treated with DAAs has been reported by many authors [13, 14]. This has multiple explanations: severity of cirrhosis before treatment, presence of risk factors for developing HCC before treatment (age > 50 years, male sex, platelets < 100,000/mm^3^), and presence of co-morbidities (alcohol, diabetes, and non-alcoholic steatohepatitis). A striking finding in our study was the role of HCV genotype. Indeed, analysis of the factors associated with the development of HCC highlighted the role of the HCV genotype 3. However, this genotype was more significantly frequently found in patients exposed to DAAs. Indeed, the persistence of HCC risk after treatment reinforces the overriding recommendation to continue the 6-month monitoring of cirrhotic patients by ultrasound to detect small curable cancers.

Another highlight of our study was the high rate of SVR (88%), even among the most severe patients (87%). These results are similar to those reported elsewhere in pivotal [3, 4] or real-life [5] studies dedicated to patients with decompensated cirrhosis. Again, what distinguishes our approach here from that of the aforementioned studies was our measure of the effects of viral eradication on our main judgment criteria beyond SVR. Indeed, our results demonstrate that viral eradication was associated with a significant better clinical outcome in terms of mortality, HCC occurrence, and need for LT. The latter is in line with the European study showing that viral eradication could allow the delisting of 20% of treated patients while on the waiting list for LT [15].

There are some limitations of our study. Firstly, 45% of patients who met the inclusion criteria, i.e. a previous history of decompensated cirrhosis prior to or at the time of study inclusion, had a Child–Pugh score of A. It is conceivable that co-morbidities that contribute to the aggravation of cirrhotic patients were managed after decompensation. For example, it can be noted that excessive alcohol consumption was only 2% in the whole population at the time of inclusion for study. In turn, due to the systemic hemodynamic alterations of cirrhosis, once a complication related to portal hypertension occurs in a cirrhotic patient, the disease is considered as decompensated [16]. A second limitation is that only 55 patients had a very severe disease, i.e. Child–Pugh score of C and/or MELD score > 20. This made statistical analysis, other than comparison of crude incidences, impossible. This is also the reason why the effects of SVR in these patients could not be analysed.

## Conclusion

In summary, this prospective cohort study involving HCV-infected patients having experienced an episode of decompensated cirrhosis illustrates an overall significant decrease in risk for all-cause mortality associated with DAA treatment. Our results also show that SVR is associated with a decrease in risk for HCC occurrence and need for LT. On this basis, DAA treatment should be considered for any patient with HCV-related decompensated cirrhosis.

## Supplementary Information

**Additional file 1: Table S1.** Balance of baseline covariates following IPTW analysis according to DAA exposure. **Table S2.** Summary of non-liver related deaths by cause. **Table S3.** Summary of liver related deaths by cause. **Table S4.** Factors associated with HCC, all-cause mortality, and liver transplant in all 559 patients included for study. **Table S5.** Characteristics of hepatocellular carcinoma according to DAA exposure. **Table S6.** Incidence rates of hepatocellular carcinoma, all-cause mortality, liver-related mortality, non-liver-related mortality, and liver transplant in all 559 patients under study according to DAA exposure period and virological response status. **Table S7.** Incidence rates of hepatocellular carcinoma, all-cause mortality, liver-related mortality, non-liver-related mortality, and liver transplant in all 55 patients with a Meld score>20 or Child-Pugh score C according to DAA exposure period and virological response status.

## Data Availability

All data generated or analysed during this study are included in this published article [and its supplementary information files].

## Abbreviations

DAAs: Direct-acting antiviral agents
HCV: Hepatitis C virus
SVR: Sustained virological response
HCC: Hepatocellular carcinoma
AHT: Arterial hypertension
LR: Liver-related
NLR: Non liver-related
LT: Liver transplantation
BMI: Body mass index
ALT: Alanine aminotransferase
AST: Aspartate aminotransferase
AFP: Alpha-fetoprotein
IPTW: Inverse probability of treatment weighting
