# Supplementary material for: Clinical outcomes after treatment with direct antiviral agents: beyond the virological response in patients with previous HCV-related decompensated cirrhosis
Source: BMC Infect Dis. 2022 Jan 27;22:94. doi: 10.1186/s12879-022-07076-0 (PMC8796427; doi:10.1186/s12879-022-07076-0)
Supplement: Supplementary file 1 — Additional file 1: Table S1. Balance of baseline covariates following IPTW analysis according to DAA exposure. Table S2. Summary of non-liver related deaths by cause. Table S3. Summary of liver related deaths by cause. Table S4. Factors associated with HCC, all-cause mortality, and liver transplant in all 559 patients included for study. Table S5. Characteristics of hepatocellular carcinoma according to DAA exposure. Table S6. Incidence rates of hepatocellular carcinoma, all-cause mortality, liver-related mortality, non-liver-related mortality, and liver transplant in all 559 patients under study according to DAA exposure period and virological response status. Table S7. Incidence rates of hepatocellular carcinoma, all-cause mortality, liver-related mortality, non-liver-related mortality, and liver transplant in all 55 patients with a Meld score>20 or Child-Pugh score C according to DAA exposure period and virological response status. [file 12879_2022_7076_MOESM1_ESM.docx]

**Table S1.** **Balance of baseline covariates following IPTW analysis according to DAA exposure.**

|  | Received DAA after study inclusion  (n=483) | Did not receive DAA after study inclusion  (n=76) | **Standardised difference (%)** |
| --- | --- | --- | --- |
| Age in years (mean ± SD) | 57·7 (± 10·1) | 57·8 (± 10·4) | -0·8 |
| Male gender (%) | 68·8 | 68·9 | -0·1 |
| BMI (kg/m^2^) (%)  <18·5  [18·5, 25[  [>25, 30[  > 30 | 3·2  46·8  34·3  15·7 | 3·2  46·8  34·2  15·8 | 0 |
| Geographic Origin (%)  Asia  Eastern Europe  France  North Africa  Other  Sub-Saharan Africa | 1·5  4·6  61·5  16·7  12·1  3·6 | 1·6  5·2  61·4  16·2  12·3  3·2 | 9·0 |
| Infection route (%)  Injecting drug use  Transfusion  Other or Unknown | 31·2  27·8  41·0 | 31·2  27·8  41·0 | 0 |
| Time since HCV diagnosis in years (mean ± SD) | 14·2 (± 8·3) | 14·3 (± 8·5) | -1·4 |
| HCV treatment history (%)  Treatment-experienced  Treatment-naive | 68  32 | 68  32 | 0·1 |
| HCV genotype (%)  1  2  3  4  5/6/7 | 58·8  5·4  21·5  13·1  1·3 | 58·7  5·4  21·6  13·0  1·3 | 0 |
| Fib4 score  (mean ± SD) | 7·7 (± 6) | 7·6 (± 6·3) | 1·6 |
| APRI score  (mean ± SD) | 2·8 (± 2·7) | 2·8 (± 3·1) | 1·3 |
| MELD score (%)  <13  [13 ; 20[  >20 | 68·9  24·5  5·4 | 65·6  27·3  5·4 | 6·9 |
| Diabetes (%)  No  Yes | 76·4  23·6 | 76·4  23·6 | -0·04 |
| Arterial hypertension (%)  No  yes | 67·6  32·4 | 67·7  32·3 | 0·1 |
| Anaemia (%)  No  Yes | 69·8  30·2 | 69·8  30·2 | 0·1 |
| Albumin (mean ± SD) | 34·9 (± 6·5) | 34·9 (± 6·3) | 0.11 |
| Prothrombin time (mean ± SD) | 73·2 (± 17·4) | 73·0 (± 17·4) | 0.93 |
| Platelet count (mean ± SD) | 106991 (± 59157) | 108892 (± 60221) | -3.18 |
| Alanine aminotransferase (mean ± SD) | 72·8 (± 52·6) | 72·2 (± 53·3) | 0.95 |
| Aspartate aminotransferase (mean ± SD) | 86·7 (± 48·1) | 90·0 (± 64·4) | -5.95 |
| Alpha-fetoprotein (mean ± SD) | 15·5 (± 31·1) | 15·6 (± 27·4) | -0.22 |
| Bilirubin (mean ± SD) | 27·2 (± 30·0) | 27·7 (± 22·3) | -1.88 |
| Child-Pugh score (%)  A  B  C | 55·9  37·7  6·4 | 54·5  37·3  8·2 | 7·9 |
| Past excessive alcohol use (%)  No  Yes | 48·6  51·4 | 48·7  51·3 | 0·1 |
| Excessive alcohol use at study inclusion (%)  No  Yes | 98  2 | 97  3 | 9·1 |
| Time since decompensation of cirrhosis in months  (mean ± SD) | 21·2 (± 19) | 19·4 (± 18·6) | 9·4 |

**Table S2. Summary of non-liver related deaths by cause**

| **System Organ (MedDRA v17.0 classification)** | **Number (cause)** |
| --- | --- |
| Neoplasm benign, malignant and unspecified | 6 (1 gastric, 2 colon, 1 lung, 1 oropharyngeal, 1 brain) |
| Cardiac disorders | 5 (2 cardiac failure, 2 myocardial infarction, 1 cardiac disorder) |
| Gastrointestinal disorders | 2 (gastric ulcer) |
| General disorders and administration site conditions | 2 (deaths of unknown origin) |
| Infections and infestations | 6 (6 sepsis) |
| Injury, poisoning, and procedural complications | 2 (1 road traffic accident, 1 head injury) |
| Nervous system disorders | 8 (6 stroke, 1 cerebral haematoma, 1 metabolic encephalopathy) |
| Psychiatric disorders | 1 (Suicide) |
| Renal and urinary disorders | 1 (Renal failure) |
| Respiratory, thoracic, and mediastinal disorders | 1 (1 pulmonary embolism) |
| Vascular disorders | 1 (haemorrhagic shock) |

**Table S3. Summary of liver related deaths by cause**

| **System Organ (MedDRA v17.0 classification)** | **Number (cause)** |
| --- | --- |
| Neoplasm benign, malignant and unspecified | 24 (HCC) |
| Gastrointestinal disorders | 11 (4 gastrointestinal haemorrhage, 6 oesophageal varice haemorrhage, 1 upper gastrointestinal haemorrhage) |
| Hepatobiliary disorders | 25 (3 ascites, 6 hepatic cirrhosis, 5 hepatic encephalopathy, 7 hepatic failure, 1 alcoholic hepatitis, 2 hepatorenal syndrome, 1 liver disorder) |
| General disorders and administration site conditions | 1 deaths of unknown origin |
| Infections and infestations | 10 (1 Escherichia sepsis, 2 peritonitis, 7 septic shock) |
| Injury, poisoning and procedural complications | 3 (1 transplant surgery complication, 1 transplanted liver complication, 1 procedural complication) |

**Table S4. Factors associated with HCC, all-cause mortality, and liver transplant in all 559 patients included for study**

| Variables | HCC  HR [95% CI] | Death  HR [95% CI] | Liver related mortality  HR [95% CI] | Liver transplant  HR [95% CI] |
| --- | --- | --- | --- | --- |
| Age in years  <50 (ref)  [50-<56[  [56-<64[  ≥64 | 1  0·77 (0·37–1·61)  1·68 (0·84–3·36)  2·04 (0·87–4·81) | 1  1·75 (0·67–4·54)  2·32 (0·83–6·52)  2·77 (0·83–9·20) | 1  3·43 (0·71–16·55)  3·02 (0·54–16·80)  5.44 (0.69-42.78) | 1  0·62 (0·21–1·82)  0·15 (0·01–1·64)  *** |
| Male gender (vs female) | 1·16 (0·61–2·22) | 0·92 (0·47–1·77) | 0·51 (0·19–1·41) | 0·95 (0·07–12·40) |
| BMI (kg/m^2^)  <18·5  [18·5, 25[ (ref)  [>25, 30[  >30 | 1·11 (0·33–3·75)  1  0·88 (0·53–1·47)  0·72 (0·36–1·46) | 0·99 (0·17–5·89)  1  1·21 (0·69–2·12)  1·34 (0·68–2·62) | ***  1  2·07 (0·86–3·99)  1·318 (0·47–3·64) | ***  1  1·20 (0·28–5·24)  0·59 (0·08–4·39) |
| Geographic Origin France (vs other) | 1·28 (0·75–2·19) | 1·00 (0·56–1·78) | 1·04 (0·46–2·34) | 3·45 (0·70–16·89) |
| Infection route  Injecting drug use (ref)  Transfusion  Other or unknown | 1  0·40 (0·18–0·90)  0·65 (0·36–1·16) | 1  0·48 (0·18–1·27)  0·87 (0·40–1·87) | 1  0·28 (0·07–1·14)  0·38 (0·09–1·59) | 1  1·94 (0·25–14·86)  1·68 (0·38–7·44) |
| Excessive alcohol use  at study inclusion (Y vs N)  Past (Y vs N) | 1·30 (0·19–9·10)  0·99 (0·58–1·71) | 172 (0·42–7·15)  1·26 (0·65–2·47) | 3.13 (0.60-16.22)  1·45 (0·50–4·18) | ***  1·01 (0·25–4·14) |
| Time since HCV diagnosis in years  <7 (ref)  [7-15[  [15-21[  ≥21 | 1  0·44 (0·22–0·88**)**  0·92 (0·48–1·76)  1·29 (0·64–2·60) | 1  0·91 (0·40–2·09)  1·14 (0·49–2·68)  0·78 (0·30–2·03) | 1  0·71 (0·16–3·05)  1·48 (0·34–6·42)  1·06 (0·22–5·07) | 1  1·34 (0·20–9·25)  0·63 (0·07–5·64)  3·01 (0·40–22·52) |
| HCV treatment-naive (Y vs N) | 0·85(0·50–1·45) | 0·83 (0·40–1·69) | 0·54 (0·15–1·94) | 1·05 (0·30–3·73) |
| HCV genotype  1 (ref)  2  3  4  5/6/7 | 1  2·94 (1·18–7·31)  2·56 (1·44–4·55)  0·99 (0·42–2·35)  2·39 (0·22–26·04) | 1  1·53 (0·53–4·41)  0·76 (0·34–1·69)  0·43 (0·17–1·13)  1·74 (0·54–5·64) | 1  2.03 (0.28-14.79)  1·35 (0·44–4·17)  0·99 (0·29–3·32)  7·84 (1·85–33·11) | 1  ***  2·97 (0·76–11·54)  1·08 (0·04–30·07)  4·95 (0·32–75·77) |
| Diabetes (Y vs N) | 0·94 (0·52–1·73) | 0·96 (0·54–1·71) | 0·97 (0·41–2·25) | 1·66 (0·29–9·44) |
| Hypertension (Y vs N) | 0·80 (0·45–1·41) | 1·78 (1·02–3·13) | 1·77 (0·70–4·46) | 2·11 (0·36–12·57) |
| Albumin (<30g/L vs ≥30g/L) | 0·90 (0·50–1·60) | 0·91 (0·45–1·81) | 0·71 (0·26–1·91) | 1·58 (0·50–5·02) |
| Prothrombin time (≤70% vs >70%) | 1·88 (1·03–3·43) | 1·42 (0·73–2·75) | 2·53 (0·90–7·12) | 3·41 (0·77–15·10) |
| Platelet count (<10^5^/µL vs ≥10^5^/µL) | 1·16 (0·68–2·00) | 1·66 (0·85–3·24) | 1·87 (0·71–4·94) | 1·47 (0·26–8·21) |
| Alanine aminotransferase (>40 UI/L vs ≤40 UI/L) | 0.98 (0.50-1.93) | 0.63 (0.34-1.17) | 0.54 (0.22-1.35) | 2.49 (0.47-13.16) |
| Aspartate aminotransferase (>40 UI/L vs ≤40 UI/L) | 2·27 (0·85–6·04) | 1·73 (0·77–3·86) | 7·59 (1·74–33·23) | 1·56 (0·12–20·70) |
| Alpha-fetoprotein (≥5·5 ng/mL vs <5·5 ng/mL) | 2·20 (1·26–3·85) | 0·90 (0·53–1·55) | 1·22 (0·52–2·89) | 1·29 (0·51–3·27) |
| Child-Pugh score  B vs A  C vs A | 1.45 (0.79-2.65)  2.96 (1.16-7.51) | 1.52 (0.75-3.09)  3.22 (0.99 – 10.49) | 4.38 (1.27-15.06)  11.33 (1.71-75.21) | 9.94 (1.58-62.65)  19.24 (2.89-128.13) |

***: Not performed or grouped with another class (Age [56-<64[, BMI [18·5, 25[, genotype 1) due to insufficient number of events

**Table S5. Characteristics of hepatocellular carcinoma according to DAA exposure.**

| **Characteristics** | **Not exposed to DAA**  **(n=20)** | **Exposed to DAA**  **(n=72)** | **P-value** |
| --- | --- | --- | --- |
| Time between last normal evaluation and first abnormal evaluation (months)  *Missing* | 13·3 ± 14·2  *5* | 8·7 ± 9·4  *22* | 0·57 |
| Time between first abnormal evaluation and diagnosis  *Missing* | 9·0 ± 22·2  *2* | 5.4 ± 11·8  *6* | 0·84 |
| Time between last normal evaluation and diagnosis  *Missing* | 19·1 ± 17·3  *5* | 15.6 ± 19·4  *20* | 0·48 |
| Macroscopic pattern  Infiltrative  Nodular  *Missing* | 2 (10%)  18 (90%)  *0* | 10 (14%)  60 (86%)  *2* | 1·00 |
| Nodular patterns:  Number of tumors at diagnosis  *Missing*  Largest nodule size (in mm)  *Missing*  Total nodule size (in mm)  *Missing* | 1·6 ± 0.9  *2*  23·4 ± 11·2  *2*  30·7 ± 21·5  *2* | 1·9 ± 1·4  *13*  25·5 ± 15·5  *12*  36·1 ± 24·8  *17* | 0·48  0·73  0·29 |
| Alpha-fetoprotein (in log(ng/mL))  at entry  *missing*  at diagnosis  *missing* | 2·3 ± 1·0  *2*  2·6 ± 1·1  *1* | 2·2 ± 1·0  *9*  3·6 ± 2·4  *10* | 0·98  0·25 |
| Liver biopsy at diagnosis  missing  Grade (WHO)  Well differentiated  Moderately differentiated  Poorly differentiated/Undifferentiated  Cholangiocarcinoma  Not interpretable  Others  Missing | 6 (32%)  *1*  3  2  0  0  0  0  15 | 23 (33%)  *2*  10  6  2  1  0  4  49 | 0·92 |

**Table S6. Incidence rates of hepatocellular carcinoma, all-cause mortality, liver-related mortality, non-liver-related mortality, and liver transplant in all 559 patients under study according to DAA exposure period and virological response status.**

|  | **Not exposed**  **N=76** | | **On treatment**  **N=483** | | **SVR**  **N=391** | | **No SVR**  **N=53** | | **Unknown SVR**  **N=39** | |
| --- | --- | --- | --- | --- | --- | --- | --- | --- | --- | --- |
|  | n/pyr | Incidence per 100 pyrs (95% CI) | n/pyr | Incidence per 100 pyrs (95% CI) | n/pyr | Incidence per 100 pyrs (95% CI) | n/pyr | Incidence per 100 pyrs (95% CI) | n/pyr | Incidence per 100 pyrs (95% CI) |
| Hepatocellular carcinoma (N=92) | 20/325 | 6·1  (3·8–9·5) | 17/277 | 6·1  (3·6–9·8) | 41/941 | 4·4  (3·1–5·9) | 11/87 | 12·6  (6·3–22·6) | 3/70 | 4·3  (0·9–12·6) |
| All-cause mortality (N=119) | 39/326 | 12·0  (8·5–16·4) | 15/276 | 5·4  (3·0–9·0) | 49/952 | 5·1  (3·8–6·8) | 10/97 | 10·3  (4·9–18·9) | 6/77 | 7·8  (2·8–16·9) |
| Liver-related mortality (N=74) | 22/326 | 6·8  (4·2–10·2) | 8/276 | 2·9  (1·3–5·7) | 33/952 | 3·5  (2·4–4·9) | 7/97 | 7·2  (2·9–14·8) | 4/77 | 5·2  (1·4–13·2) |
| Non-liver-related mortality (N=35) | 11/326 | 3·4  (1·7–6·0) | 5/276 | 1·8  (0·6–4·2) | 15/952 | 1·6  (0·9–2·6) | 2/97 | 2·1  (0·2–7·4) | 2/77 | 2·6  (0·3–9·3) |
| Liver transplant (N=36) | 10/327 | 3·1  (1·5–5·6) | 8/278 | 2·9  (1·2–5·7) | 12/955 | 1·3  (0·6–2·2) | 4/97 | 4·1  (1·1–10·5) | 2/79 | 2·5  (0·3–9·2) |

**Table S7. Incidence rates of hepatocellular carcinoma, all-cause mortality, liver-related mortality, non-liver-related mortality, and liver transplant in all 55 patients with a Meld score>20 or Child-Pugh score C according to DAA exposure period and virological response status.**

|  | **Not exposed**  **N=12** | | **On treatment**  **N=43** | | **SVR**  **N=33** | | **No SVR**  **N=5** | | **Unknown SVR**  **N=5** | |
| --- | --- | --- | --- | --- | --- | --- | --- | --- | --- | --- |
|  | n/pyr | Incidence per 100 pyrs (95% CI) | n/pyr | Incidence per 100 pyrs (95% CI) | n/pyr | Incidence per 100 pyrs (95% CI) | n/pyr | Incidence per 100 pyrs (95% CI) | n/pyr | Incidence per 100 pyrs (95% CI) |
| Hepatocellular carcinoma (N=11) | 7/22 | 31·2 (12·5-64.3) | 2/24 | 8·4 (1·0-30·5) | 1/66 | 1·5 (0·0-8·4) | 0/3 | 0·0 (0·0-121·7) | 1/7 | 14·6 (0·4-81·4) |
| All-cause mortality (N=20) | 10/20 | 49·7 (23·8-91·3) | 2/24 | 8·3 (1·0-30·1) | 7/61 | 11·5 (4·6-23·7) | 0/1 | 0·0 (0·0-335·6) | 1/7 | 15·2 (0·4-84·7) |
| Liver-related mortality (N=13) | 6/20 | 29·8 (10·9-64·8) | 2/24 | 8·3 (1·0-30·1) | 4/61 | 6·6 (1·8-16·8) | 0/1 | 0·0 (0·0-335·7) | 1/7 | 15·2 (0·4-84·7) |
| Non-liver-related mortality (N=5) | 2/20 | 9·9 (1·2-35·9) | 0/24 | 0·0 (0·0-15·4) | 3/61 | 4·9 (1·0-14·4) | 0/1 | 0·0 (0·0-335·7) | 0/7 | 0·0 (0·0-56·1) |
| Liver transplant (N=9) | 4/20 | 20·0 (5·5-51·3) | 2/24 | 8·4 (1·0-30·4) | 2/59 | 3·4 (0·4-12·2) | 1/1 | 91·0 (2·3-507·0) | 0/7 | 0·0 (0·0-56·0) |
